# Macrophages assemble! But do they need IL‐4R during schistosomiasis?

**DOI:** 10.1002/eji.201948158

**Published:** 2019-07-03

**Authors:** Dominik Rückerl, Peter C. Cook

**Affiliations:** ^1^ Lydia Becker Institute of Immunology and Inflammation Faculty of Biology Medicine and Health University of Manchester Manchester Academic Health Science Centre Manchester UK; ^2^ Lydia Becker Institute of Immunology and Inflammation Manchester Collaborative Centre for Inflammation Research Faculty of Biology Medicine and Health University of Manchester Manchester Academic Health Science Centre Manchester UK

**Keywords:** IL‐4, liver, macrophages, monocytes, Schistosoma mansoni

## Abstract

Helminth infections are a global health burden in humans and livestock and are considered to be a major evolutionary driver of type 2 immunity (orchestrated by type 2 cytokines, e.g., IL‐4 and IL‐13). Upon infection, helminths cause substantial damage to mucosal tissues as they migrate within the host and elicit crucial protective immune mechanisms. Macrophages, essential innate cells, are known to adopt a specific activation status (termed M(IL‐4)) in type 2 cytokine environments. Yet, the role of these macrophages in mediating protective immune/wound healing responses to helminths is unclear. Furthermore, macrophage subsets can be very heterogenous (linked to their differing cellular origins) and the relative role of these subsets in the context of M(IL‐4) activation to helminth infection is unknown. An article by Rolot *et al*. in this issue of the *European Journal of Immunology* [Eur. J. Immunol. 2019. 49: 1067–1081] uses a variety of transgenic murine strains to revise our understanding of the complexity of how these subsets undergo M(IL‐4) activation and participate in wound healing responses in helminth infection. Here we highlight that consideration of different macrophage subsets in mucosal tissues is essential when evaluating the functional role of M(IL‐4) macrophages.

Helminths, a huge global burden on public health, induce potent type 2 and immuno‐regulatory networks which can limit the damage these parasites cause 1. Therefore, studying the immune response during helminth infection and potentially exploiting this information for the development of novel therapeutics has become a field of great interest to the scientific community. A hallmark of anti‐parasite type 2 immunity is the IL‐4 receptor (IL‐4Rα, essential for IL‐4 and IL‐13 downstream signaling) mediated activation of macrophages and the resulting M(IL‐4) activation phenotype, which has been associated with many crucial features of resistance to infection 2. M(IL‐4) activation has been proposed to play a central role in inducing rapid wound repair, necessary due to the large physical damage that helminths can cause 3. Furthermore, M(IL‐4) macrophages can accumulate through proliferative expansion of tissue resident cells avoiding excessive inflammation and associated pathology 4, 5. In the current issue of the *European Journal of Immunology* Rolot et al. have re‐evaluated the role of IL‐4R expression on macrophages in a rodent model of *Schistosoma mansoni* infection (a potent inducer of type 2 immunity) and have found a limited role for M(IL‐4) activation in the recruitment of macrophages, the resistance to infection or survival of experimental animals 6. Macrophages in the liver of animals infected with *S. mansoni* were largely derived from blood monocytes, which accumulated independently of IL‐4Rα‐mediated signals, whereas tissue resident macrophage numbers declined simultaneously. M(IL‐4) activation (i.e., Relm‐α, Ym1, Arg1) was predominantly evident in monocyte‐derived cells, as compared to resident Kupffer cells, but seemed dispensable for resistance to *S. mansoni* infection. Last, depletion of tissue resident macrophages during infection had no impact on the outcome of the infection (findings summarized in Fig. 1). The work by Rolot et al. highlights the complexity of macrophage recruitment and activation during type 2 inflammation in intricate immunological environments.

**Figure 1:**
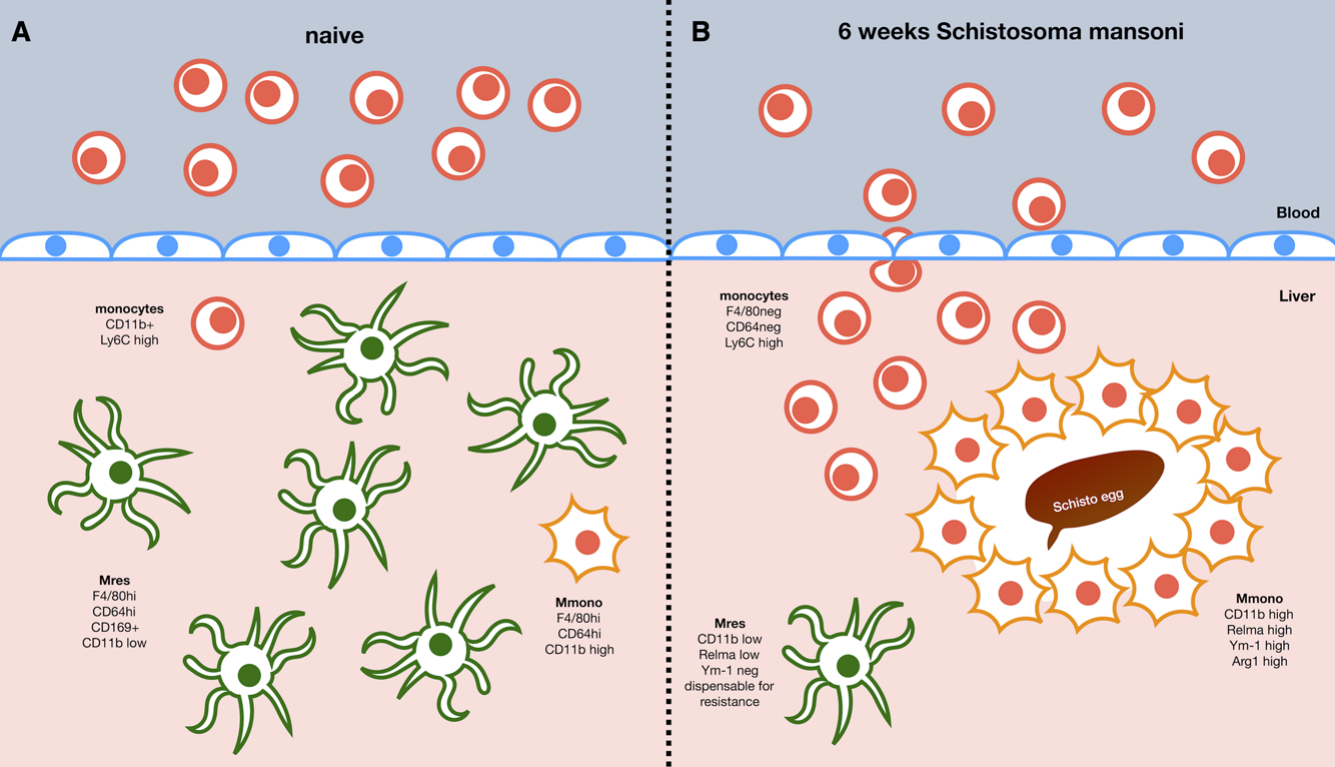
Macrophage populations in the liver in (A) naive animals or (B) 6 weeks after *Schistosoma mansoni* infection. In naive animals, the majority of macrophages display a resident phenotype (CD11b low) with only a minority of monocytes (Ly6C high) and monocyte derived macrophages (Mmono, CD11b high) detectable. *S.mansoni* infection leads to a pronounced influx of Ly6C high monocytes differentiating into CD11b hi Mmono whereas resident Kupffer cells undergo a pronounced Macrophage Disappearance Reaction, potentially due to enhanced cell death. IL‐4Rα signaling to macrophages plays no role in the accumulation dynamics but induces markers of M(IL‐4) activation (i.e. Relm‐α, Ym1 or Arginase 1) particularly in Mmono but almost none in Mres. Whether resident CD11b low macrophages contribute to the granuloma formation in schistosome infections is currently unclear.

Helminth infection typically involves migration of larvae within a mammalian host across several tissue barriers, which can cause substantial physical damage 3. A central paradigm of M(IL‐4) activation has been that these macrophages are critical for mediating wound repair. This is supported by the observation that IL‐4R expressing macrophages are critical for collagen fibril assembly in skin repair 7 as well as following myocardial infarction 8. In chronic *S. mansoni* infection, both in humans and mice, parasite eggs become trapped in the liver and other organs leading to a granulomatous response and fibrosis governed by type 2 immunity. Macrophages have been shown to be a key component for granuloma formation, which is an essential aspect of the anti‐parasite response that prevents host mortality 9. Yet conflicting results have emerged on whether macrophages require IL‐4Rα to enable these protective responses. Restriction of IL‐4Rα deficiency to lysozyme M (LysM) expressing cells (a feature of macrophages and neutrophils) was observed to lead to larger, less dense granulomas and a higher mortality 10. However, a more recent study found that macrophage specific IL‐4Rα deficiency led to enhanced liver inflammation but observed no impact on mortality or systemic pathology 11. In their manuscript Rolot et al. turned their attention to this unresolved issue and in agreement with Vanella et al. found heightened liver granuloma pathology, but not mortality, in infected IL‐4Rα^flox/−^LysM^Cre^ mice. Simultaneously, M(IL‐4) activation was strongly diminished, albeit not completely abolished 6. This raises the question whether M(IL‐4) activity is indeed important for wound healing during *S. mansoni* infection. However, as highlighted by Rolot et al. these observations relied on LysM‐driven Cre expression as a method to deplete IL‐4Rα expression and the authors highlight that a significant proportion of macrophages and particularly monocytes were not expressing LysM. This demonstrates that new transgenic approaches to reliably deplete IL‐4Rα expression on all macrophages and monocytes are required to fully evaluate the systemic function of IL‐4 activation of macrophages during *S. mansoni* infection.

The paradigm that accumulation of macrophages at the site of infection was due to recruitment of bone‐marrow derived blood monocytes from the vasculature 12 was challenged approximately 10 years ago and it is now clear that multiple mechanisms can give rise to macrophage populations dependent on the physiological context. Bacterial infections and pro‐inflammatory/type 1 mediated conditions in general, largely induce influx of monocytes from the vasculature which mature and activate into macrophages in situ 13. In contrast, during type 2 responses to helminth infection, high levels of IL‐4 in the tissue environment have been shown to drive the proliferative expansion of tissue resident macrophages (Mres) as well as monocyte derived macrophages (Mmono) if they are present in the tissue 4, 5. However, this dichotomy of recruitment mechanisms aligning with the type of immune response (i.e., type 1/type 2) is overly simplified. In particular, *S. mansoni* infection triggers recruitment of monocyte derived macrophages, which become M(IL‐4) activated in situ 9, 14. Nonetheless, it was assumed IL‐4 signaling is still important to further expand and maintain the recruited population 2. Rolot et al. now show that the influx of blood monocytes and their conversion to macrophages in the liver is completely independent of IL‐4Rα expression by macrophages (Fig 1). Indeed, the authors found limited evidence that proliferative expansion in general, let alone driven by IL‐4, contributes to the number of macrophages present in the liver at any stage during the infection 6. Thus, despite the prevalent type 2 environment during *S. mansoni* infection, IL‐4Rα mediated signals do not appear to be involved in the expansion/recruitment of macrophages in the liver. It is worth to note, that IL‐4 driven expansion of Mres requires very high doses of IL‐4 as well as tissue‐specific co‐factors 15, which may not be present in the liver during *S. mansoni* infection. Moreover, whether Mres are involved in granuloma formation and thus exposed to the necessary doses of IL‐4 remains to be determined (Fig 1). In addition, even in helminth models with dominant expansion of tissue resident macrophages other factors, like CSF‐1, have been shown to be crucial for the initial increase in cell numbers 4. IL‐4 may thus signal to other cells during *S.mansoni* infection and indirectly support the expanded population of macrophages akin to an increased number of tissue niches 16. In this context it is important to notice, that a recent study found fundamental differences in the capacity of two very common laboratory mouse strains (i.e., C57BL/6 vs. BALB/c) to maintain Mres and Mmono populations during chronic helminth infection 17. Moreover, macrophage populations are not static and changes in composition continuously occur. A multitude of factors including affected tissue, type of immune response, sex, age and genotype of the host as well as length of time post initial infection determine the composition of macrophage populations during helminth infection. Therefore, it will now be of great interest to see whether the differences in cellular composition translate into functional differences and thus whether the role of macrophages changes over the course of an infection.

In the last part of their manuscript Rolot et al. turned their attention to the role of Mres in the resistance to *S. mansoni* infection. Their data showed a marked loss in the number of these cells over the course of the infection as well as rather limited induction of M(IL‐4) markers (Fig 1) 6. This indicated that Mres might not be involved in the immune response against *S. mansoni*. Indeed, depletion of liver Mres using a CD169‐DTR model resulted in unaltered survival and granuloma formation as well as largely unchanged cellular infiltration to the liver 6. This is not limited to the liver, as it has been recently demonstrated that alveolar macrophages (i.e. lung Mres) displayed muted M(IL‐4) activation profiles compared to Mmono during *Nippostrongylus brasiliensis* infection 18. Furthermore, this discrepancy was linked to different metabolic programs in Mres as compared to Mmono 18. Similar differences in metabolism linked to an altered response profile of alveolar macrophages have also been observed in a model of acute lung inflammation and injury 19. Thus, taken together with data obtained using peritoneal macrophages 20, 21, this suggests, that the developmental origin of a macrophage maybe as important as the immediate cytokine milieu for governing M(IL‐4) activation. Therefore, preferential recruitment and activation of Mmono in *S. mansoni* infection may indicate a requirement for a different “M(IL‐4)‐response” than helminth infections triggering Mres expansion (e.g. *L. sigmodontis*). At the moment it is unclear what the differences of these responses are and how this contributes to the resistance to each particular helminth.

An explanation may lie within the affected tissue itself. Mres are seeded to virtually every tissue during embryonic development and are then maintained locally via different mechanisms, depending on the tissue as well as the age of the mouse 22. All Mres share a set of core genes confirming their identity as macrophages (e.g. lysosomal biogenesis). However, on top of this, every tissue imprints a unique signature imparting tissue identity and equipping Mres to tackle tissue specific tasks (e.g. bone resorption by osteoclasts) 23. This tissue specific imprinting renders some Mres populations unresponsive (e.g. alveolar macrophages) and leaves others responsive (e.g. pleural macrophages) 18. Thus, depending on which organ is affected by the helminth infection, will determine whether the macrophage response is dominated by local proliferation of Mres or recruitment of Mmono. It is important to note, though, that this imprinting is reversible, and Mres taken out of their environment will regain their responsiveness 18, 24. This shows that care must be taken when interpreting results from tissue macrophages that have been generated after they have been removed from their environment. Moreover, it is feasible to imagine, that there may be circumstances under which Mres in the lung or liver become the dominant macrophage population responding to a challenge.

The loss of Mres described by Rolot et al. is a well‐known phenomenon during inflammatory conditions known as the macrophage disappearance reaction 25. It affects any type of Mres in any tissue and has been proposed to protect the host from intracellular pathogens, that can hijack Mres, due to the metabolic differences discussed above, and propagate inside these cells as ideal hosts 26. Thus, during helminth infection the macrophage disappearance reaction may seem obsolete. But potentially it reflects competition for suitable tissue niches between Mres and Mmono 21, 26. The disappearance of Mres may, thus, allow the long‐term survival of recruited Mmono and ensure a lasting anti‐helminth response. Rolot et al. conclude from their data that Mres have no role in the resistance to *S. mansoni* infection. However, it has to be noted, as also shown by the authors, that Mres have a clear anti‐inflammatory role, limiting the influx of neutrophils and monocytes 6 and regulating the silent removal of apoptotic cells under steady state 27. Thus, although the authors did not observe any effects of Mres depletion on survival in their model, Mres may play an important role in avoiding immunopathology in chronic human infections, or may be a deciding factor distinguishing patients who develop symptoms of the disease or stay asymptomatic 28.

Overall the work of Rolot et al. provides a much‐needed insight into the role of IL‐4Rα expressing macrophage subsets in the liver which mediate protective responses during helminth infection. This work highlights the complexity of macrophage responses to type‐2 cytokine environments. Therefore, as M(IL‐4) populations have been located in a variety of tissues and inflammatory situations (e.g. parasite infection, allergy and cancer) this study shows that careful functional in situ assessment of these populations is essential.

## Conflict of interest

The authors declare no financial or commercial conflict of interest.

**Abbreviation**
LysM: lysozyme M
